# Low Prevalence of Iron and Vitamin A Deficiency among Cambodian Women of Reproductive Age

**DOI:** 10.3390/nu8040197

**Published:** 2016-04-01

**Authors:** Frank T. Wieringa, Prak Sophonneary, Sophie Whitney, Bunsoth Mao, Jacques Berger, Joel Conkle, Marjoleine A. Dijkhuizen, Arnaud Laillou

**Affiliations:** 1 UMR-204 Nutripass, Institut de Recherche pour le Développement, IRD/UM/SupAgro, 34394 Montpellier, France; Jacques.berger@ird.fr; 2 National Nutrition Program, Ministry of Health, Phnom Penh, Cambodia; sophonprak@gmail.com; 3 World Food Program, Phnom Penh, Cambodia; Marie-Sophie.Whitney@echofield.eu; 4 Planning Unit, University of Health Sciences, Phnom Penh, Cambodia, maobunsoth888@yahoo.com; 5 UNICEF, Phnom Penh, Cambodia; joel.conkle@emory.edu (J.C.); alaillou@unicef.org (A.L.); 6 Laney Graduate School, Emory University, Atlanta, GA 30307, USA; 7 Department of Nutrition, Exercise and Sports, Copenhagen University, 1958 Frederiksberg C, Denmark; madijkhuizen@gmail.com

**Keywords:** iron, vitamin A, deficiency, Cambodia, women of reproductive age, inflammation

## Abstract

Nearly half of women of reproductive age (WRA) in Cambodia are anemic. To guide interventions, national data on nutritional causes of anemia, including iron deficiency and vitamin A deficiency, are needed. In 2012, a national household survey in WRA on antibodies to routine vaccine-preventable disease immunity was performed. We used serum samples from this survey to estimate the prevalence of iron and vitamin A deficiency in 2112 Cambodian WRA, aged 15 to 39 years. Iron deficiency was classified as low or marginal iron stores (ferritin concentrations corrected for inflammation <15 μg/L and <50 μg/L respectively; Fer), iron deficient erythropoiesis (soluble transferrin receptor concentrations >8.3 mg/L; sTfR), or low total body iron (TBI) derived from Fer and sTfR concentrations (<0 mg/kg). Vitamin A status was classified using retinol binding protein (RBP) concentrations corrected for inflammation as deficient (<0.70 μmol/L) or marginal (<1.05 μmol/L. Overall, the prevalence of low iron stores, low TBI and iron deficient erythropoiesis was 8.1%, 5.0% and 9.3% respectively. Almost 40% of the women had marginal iron stores. Iron status was better in women living in urban areas compared to rural areas (*p* < 0.05 for TBI and sTfR). The prevalence of vitamin A deficiency was <1%. These findings suggest that the contribution of iron and vitamin A deficiency to the high prevalence of anemia in Cambodian WRA may be limited. The etiology of anemia in Cambodia needs to be elucidated further to guide current policies on anemia.

## 1. Introduction

Iron deficiency is considered to be the most prevalent micronutrient deficiency worldwide, with over 2 billion people affected [1]. Nationally representative data on iron deficiency are scarce, especially in developing countries; hence the prevalence of iron deficiency is often estimated using anemia prevalence as proxy indicator. Although anemia can result from different etiologies, both nutritional and non-nutritional, iron deficiency is the leading cause globally [2]. It is often assumed that 30%–40% of the subjects with iron deficiency are also anemic, but causes of anemia vary widely by age, sex and geography [1,2]. Iron deficiency alone, even without anemia, affects cognitive function and neurodevelopment of infants and children [3,4,5] and iron deficiency anemia in women has been associated with reduced work capacity and increased prevalence of pregnancy complications including low birth weight [6]. Conversely, in certain populations, malaria and inflammation could be more important etiologies of anemia than iron deficiency [7]. Also, other micronutrient deficiencies, such as vitamin A, folic acid or vitamin B12 deficiency could lead to anemia [8,9]. Furthermore, populations in Africa and Southeast Asia where hemoglobinopathies (e.g., thalassemia) are relatively common [10] have a high prevalence of anemia that is unlikely to respond to iron interventions [11].

In Cambodia, the 5-yearly Demographic Health Surveys have consistently shown a high prevalence of anemia among women of reproductive age (WRA) and young children. In 2005 and 2010, 62% and 55% of the children <5 years old were anemic, respectively. For WRA, these figures were 47% and 40% [12]. One would assume that iron deficiency is highly prevalent in Cambodia, but national data are lacking. Surprisingly, a 2014 survey in WRA in one Cambodian province found a prevalence of iron deficiency of only 2% (ferritin concentrations <15 μg/L), whereas almost 30% of the 420 women were anemic (hemoglobin concentration <120 g/L) [13]. Recently, we reported similar findings for school-aged children in a different province of Cambodia, with <2% of school-aged children having low ferritin concentrations, despite an anemia prevalence of almost 16% [3]. Based on the high prevalence of anemia in WRA, and assuming iron deficiency as the major cause, the Ministry of Health of Cambodia has set iron and folic acid supplements for pregnant women as one of the top priorities for Cambodia, and is investigating ways to improve iron status of WRA. However, if anemia among Cambodian WRA is caused by factors other than iron deficiency, programs should either target those factors, or adjust its' targets in the situation where causes are not amenable to interventions, as is the case with genetic blood disorders.

To guide the Ministry of Health, national representative data on iron status in WRA are needed. In 2012, the US Centers for Disease Control and Prevention (CDC), in collaboration with the University of Health Sciences and the Ministry of Health Cambodia conducted a nationwide serological survey in WRA on immunity to polio, rubella, tetanus and measles [14]. We obtained permission from CDC and the Ministry of Health, Cambodia to analyze residual samples from this survey for iron (ferritin and soluble transferrin receptor concentrations) and vitamin A (retinol binding protein concentrations) status. Here we report nationwide data on the prevalence of iron and vitamin A deficiency in Cambodia in women aged 15–39 years.

## 2. Experimental Section

### 2.1. Selection of Subjects and Data Collection

In November and December 2012, a nationwide cross-sectional, household serological survey was conducted among women aged 15–39 years in Cambodia. The survey design and sample size were selected to provide national estimates by 5-year age strata; a detailed description of subject selection is presented elsewhere [14]. In short, 20 enumeration areas (EAs) from the 2010 Cambodia Demographic Health surveys were sampled by probability proportional to size (PPS) based on the estimated number of women aged 15–39 years in the 2008 Cambodia General Population Census. Twenty EAs were selected from each of four regions and also from Phnom Penh. In each selected EA, 22 households (HHs) were selected by systematic sampling using previously described survey methods [15]. All eligible non-pregnant women in selected HHs were invited to participate, and teams revisited HHs up to three times to seek enrolment. A target sample size of 400 women within each age-stratum (15–19, 20–24, 25–29, 30–34, 35–39) was calculated based on a desired precision of ±5% with 95% probability of achieving that precision, a design effect of 1.5, and a 10% non-response rate based on results from previous surveys.

### 2.2. Field Procedures

Field teams enrolled eligible women after providing written information about the survey and obtaining consent from participants for venous blood collection, including storage and potential future testing of blood. A brief questionnaire, including demographic information was completed, and 5 mL of blood was obtained by venipuncture. Serum was stored at 4–8 °C and transported to the National Institute of Public Health (NIPH) laboratory in Phnom Penh within 96 h of collection. In Phnom Penh, samples were centrifuged and aliquoted into two cryovials. The cryovials used in the present study were stored at −80 °C until shipped by air on dry ice to CDC, Atlanta, USA. After determination of antibodies for vaccine-preventable disease (measles, rubella, polio and tetanus), residual samples were sent to the VitMin Laboratory in Germany for the analysis of iron and vitamin A status and inflammatory proteins using a novel sandwich ELISA technique [16].

### 2.3. Micronutrient Status Determination

For the determination of iron status, two biomarkers were used: ferritin (Fer) and soluble transferrin receptor (sTfR). Fer is considered a measure of iron storage, mainly in the liver and the spleen with ~2/3 of iron stores being in the form of ferritin [17], whereas sTfR is considered a measure of tissue iron needs [18]; using both indicators, total body iron (TBI) can be estimated using the formula from Cook: body iron (mg/kg) = − (log10 (sTfR/Fer) − 2.8229)/0.1207, with both sTfR and Fer expressed in mg/L [19]. For vitamin A status, retinol-binding protein (RBP) was used. RBP is the transport protein of retinol in the circulation and can be used as a proxy indicator for retinol concentrations [20,21]. Many indicators of micronutrient status are affected by inflammation, including ferritin and RBP concentrations [22]. Therefore, to be able to correct for the effects of inflammation on these indicators, C-reactive protein (CRP) and α1-acidglycoprotein (AGP) were also measured in all individuals. Inflammation was defined as CRP >5 mg/L and/or AGP >1 g/L. Inflammation status was then categorized in four groups based on CRP and AGP levels: no inflammation (normal CRP and AGP), incubation (high CRP and normal AGP), early convalescence (high CRP and AGP), and late convalescence (normal CRP and high AGP). Correction factors were then applied to adjust values of Fer and RBP as described previously [23,24]. Correction factors for ferrtin (ferritin corrected = Fer/correction factor) were 1.30, 1.90 and 1.36 for incubation, early convalescence and late convalescence respectively. Correction factors for RBP were 0.87, 0.76 and 0.89 for incubation, early convalescence and late convalescence respectively. Cut-offs used for micronutrient status were as follows: low iron stores was defined as Fer_corrected <15 μg/L [1]; iron-deficient erythropoiesis was defined as a sTfR >8.3 mg/L [25]; total body iron <0 mg Fe/kg body weight was regarded as iron deficiency [25]. For calculation of total body iron, corrected values of ferritin were used. Vitamin A deficiency was defined as RBP_corrected <0.70 μmol/L [20]. Marginal iron status was defined as a ferritin corrected <50 μg/L [26] (including those with a ferritin concentration <15 μg/L), and marginal vitamin A status as a RBP corrected <1.05 μmol/L) [27].

### 2.4. Ethical Approval

The serosurvey protocol was approved by the National Ethics Committee for Health Research, Cambodian Ministry of Health (057 NECHR) and was approved by CDC as a public health program evaluation.

### 2.5. Statistical Analysis

Data were checked for normality using Kolmogorov-Smirnoff test. Comparisons between regions and urban/rural for continuous variables were done by Analysis of Covariance (ANCOVA), controlling for age, and including the weighing factor to allow for the sampling of the subjects. Sampling weights were calculated to take into account each stage of selection, including the sampling probability in the original DHS EA selection. Prevalence of deficiency between regions or urban/rural was analyzed using binary logistic analysis, correcting for age and sampling procedures. *p*-values < 0.05 were considered statistical significant. Data were analyzed using SPSS software (Version 20.0, IBM, New York, NY, USA).

## 3. Results

Out of 2154 women participating in the original study, spare serum samples were available for 2112 women, allowing determination of iron and vitamin A status. The mean age of the women was 26.4 years, ranging from 15 to 39 years. Of the women, 30.4% lived in an urban area whereas 69.6% of the women lived in rural areas (Table 1). Almost 15% of the women had one or more elevated acute phase proteins, signifying inflammation (Table 1). Overall, the prevalence of low iron stores (Fer < 15 μg/L) status was 8.1% (Table 1). Evidence of iron deficient erythropoiesis was found in 9.3% of the women. Over a third of the women had marginal iron stores (Fer < 50 μg/L). Total body iron was negative (signifying a deficit in iron) in 5% of the women. Women living in rural areas had slightly lower iron stores, which were reflected in a higher prevalence of women with a negative TBI and iron deficient erythropoiesis. The effect of inflammation on the estimates of the prevalence of deficiency was only minimal (Table 1).

The prevalence of iron deficiency based on low serum Fer varied considerably among regions, with the prevalence of low iron stores being the highest in the north region (11.3%) and the lowest in Phnom Penh, the South-East and South-West of Cambodia (Table 2), although the differences did not reach statistical significance (*p* = 0.10). Marginal iron stores were significantly more common in the North and West regions of Cambodia (44.0% and 44.8% respectively) as compared to the Phnom Penh (32.7%; *p* < 0.05). The prevalence of iron-deficient erythropoeisis was also significantly higher in the North than in all other regions (*p* < 0.05, Table 2).

Vitamin A deficiency (RBP < 0.70 μmol/L) was found in only 0.6% of the women (Table 1). There was no difference between urban or rural women with regard to the prevalence of vitamin A deficiency, but women in rural areas were more likely to have marginal vitamin A status than women living in urban areas (6.5% *vs.* 2.9%, *p* = 0.003). Prevalence of marginal vitamin A status was lowest in Phnom Penh, but differences among regions did not reach statistical significance (*p* = 0.13, Table 2).

## 4. Discussion

Iron deficiency has been considered a major health problem in Cambodia given the high prevalence of anemia in children <5 years of age and WRA, estimated to be 55.5% and 45.4% respectively in the most recent (2014) Demographic Health Survey. The World Health Organization estimates that on average there are 2–5 times more people with iron deficiency, than with iron-deficiency anemia in a given population, given that iron-deficiency anemia is the last stage of iron deficiency [1]. However, as the results of the present study show, iron deficiency may not be a major issue among Cambodian WRA. In our national sample of women aged 15 to 39 years, 8% had low iron stores, based on serum ferritin as the indicator and corrected for inflammation. Using TBI, an indicator of available iron, 5% of the women were deficient in iron. This poses questions on the etiology of anemia in Cambodia. Although malaria is still present in some provinces in Cambodia, the overall prevalence is low. Clearly for Cambodia, both nutritional causes such as vitamin B12 or folate deficiency, and non-nutritional such as hemoglobinopathies, may be more important causes of anemia than iron deficiency. Nationally representative data on vitamin B12 or folate status are missing but urgently needed to ascertain the cause of the high prevalence of anemia in Cambodia with almost 40% of women of reproductive age, and over 50% of children <5 years of age being anemic according to the 2014 Cambodian Demographic Health Survey [12]. In a previous study of Cambodian school children, hemoglobinopathies, especially hemoglobinopathy E, was a more important factor for anemia than vitamin A and iron status [3,28].

In contrast to iron deficiency, marginal iron status was far more common, and in some regions almost half of the women had ferritin concentrations <50 μg/L. This is concerning, as iron requirements during pregnancy increase considerably and iron deficiency in pregnancy is associated with pre-term delivery, low birth weight and iron deficiency in infancy [6]. Milman *et al.*, estimated that overall net loss during 9 mo pregnancy and delivery is around 630 mg of iron [26]. Hence, women should enter pregnancy with adequate iron stores, as maintaining iron balance from food sources is highly unlikely [26]. A ferritin concentration of 50 μg/L corresponds to ~350–400 mg of mobilizable iron [19]. In the present study almost 40% of the women did not have this minimum amount of iron stores, and are thus likely to become iron deficient if pregnancy occurred.

In Cambodia, over 70% of the pregnant women receive standard iron and folic acid supplements during pregnancy and after delivery through the national health system [12]. In the last 10 years, Cambodia has also piloted weekly iron and folic acid supplementation for WRA. However, despite evidence for an impact on iron status during pregnancy, factors such as cost and need for behavioral change have hampered implementing this policy on a large scale. In contrast, food fortification efforts, such as iron-fortified fish sauce, are currently underway [29]. These interventions have the potential to improve the iron status of WRA in an effective and cost-effective manner [30]. Importantly, to provide evidence for policy makers on effectiveness of current interventions, repetition of the current survey in a few years from now, would give evidence on whether the iron status of WRA has improved after the introduction of fortified fish sauce in Cambodia [29].

Vitamin A deficiency or marginal vitamin A status was not a major concern in Cambodia, with only 6.4% of the women having a marginal or deficient vitamin A status. Earlier, we showed that vitamin A deficiency was also not a serious health concern in ~2500 school children in one Cambodian province, with only 0.7% of children with frank vitamin A deficiency and 7.9% with marginal vitamin A status, which is similar to the vitamin A status in WRA presented in the present study [3]. In the latter study, although vitamin A deficiency was not a significant health problem, it was significantly associated with anemia [3]. In contrast to these recent studies, a survey in 2000 showed a 2% prevalence of night blindness, double of the 1% cut-off set by the International Vitamin A consultancy group, leading to the assumption that vitamin A deficiency was prevalent in Cambodia [31]. No national data on vitamin A status have been collected in the 14 years between the survey in 2000 on night blindness and the current study, hence we can only conclude that probably the most likely explanation for this difference in vitamin A deficiency prevalence is that the vitamin A status of the Cambodian population has increased considerably during the last decade.

Unfortunately, our study could only provide information on iron status and vitamin A status through the measurement of several proteins in spare serum samples, and we have no information on the anemia prevalence in our study. Moreover, we have no data on the prevalence of hemoglobinopathies in the study population, although this is likely to be high and a major contributing factor to the high prevalence of anemia. Indicators of iron status such as ferritin and sTfR have been shown to be less reliable in the Cambodian context, perhaps due to the high prevalence of hemoglobinopathies [28]. Also, RBP is a proxy indicator for retinol concentrations, and although the correlation between retinol and RBP is high, as retinol is bound to RBP in a 1:1 ratio, RBP concentrations tend to underestimate vitamin A deficiency due to the presence of holo-RBP (RBP without retinol) in the circulation, which is especially the case at lower retinol concentrations [20]. However, as the prevalence of RBP < 1.05 μmol/L is low also (<5%), we are confident that our finding of a low prevalence of vitamin A deficiency in this population is valid.

Both ferritin and RBP concentrations are known to be affected by inflammation, even in sub-clinical infection [23,24], resulting in under- (for iron status) or over-estimation (for vitamin A status) of the prevalence of deficiency [22]. In our cohort of women, the prevalence of sub-clinical inflammation was low, with less than 15% of the women affected. Moreover, we corrected the values of Fer and RBP for inflammation, leading to only slightly changed estimates of the prevalence of low iron stores (+0.6%) or vitamin A deficiency (−0.1%). Hence, it is unlikely that inflammation affected the estimate of the prevalence of iron deficiency in the present study.

## 5. Conclusions

This national survey of over 2000 WRA showed that neither iron deficiency nor vitamin A deficiency were prevalent in Cambodian women, with <10% of the women having iron deficiency, regardless of the indicator used, and <1% having vitamin A deficiency. However, marginal or low iron status affected >1/3 of the women overall, was more common in rural areas, affected almost half of the WRA in the west and north regions of Cambodia. This is a health concern given the high iron needs during pregnancy. Interventions such as iron-fortified foods are needed to improve overall iron status in WRA in Cambodia. Vitamin A status has considerably improved over the last decade and marginal vitamin A status appears not to be a major health concern in Cambodian women currently. The contribution of iron and vitamin A deficiency to the high prevalence of anemia in Cambodian WRA seems to be limited, and the etiology of anemia in Cambodia needs to be elucidated to guide current policies on anemia.

## Figures and Tables

**Table 1:** Iron, vitamin A and inflammation status of Cambodian Women of Reproductive age by urban or rural living conditions.

Indicator	All Women	Urban	Rural	*p*-Value ^4^
*N* (% total)	2112 (100%)	643 (30.4%)	1469 (69.6%)	*-*
Age (years mean ± SD)	26.4 (± 6.9)	25.9 (± 6.8)	26.6 (± 6.9)	*0.001*
Uncorrected Ferritin (μg/L; median IQR)	67.9 (35.8–108.0)	75.6 (38.2–116.0 )	63.8 (34.9–105.0)	*0.003*
Fer < 15μg/L (%) ^2^	7.5%	6.5%	8.4%	*0.18*
Fer < 50 μg/L (%) ^2^	37.9%	36.2%	39.6%	*0.19*
Corrected ^1^ Ferritin (μg/L; median IQR)	64.4 (34.8–101.4)	71.5 (37.2–107.2)	61.1 (34.4–96.5)	*<0.001*
Fer < 15μg/L (%) ^2^	8.1%	7.3%	8.8%	*0.28*
Fer < 50 μg/L (%) ^2^	39.3%	38.1%	41.8%	*0.15*
sTfR (mg/L; median IQR)	5.5 (4.7–6.7)	5.3 (4.6–6.2)	5.6 (4.8–6.8)	*<0.001*
sTfR > 8.3 mg/L (%) ^2^	9.3%	7.2%	11.5%	*0.007*
TBI (mg/kg body wt; median)	7.6 (5.0–9.5)	8.2 (5.5–9.9)	7.3 (4.8–9.3)	*<0.001*
TBI < 0 (%) ^2^	5.0%	3.7%	6.4%	*0.026*
Uncorrected RBP (μmol/L; median IQR)	1.65 (1.36–2.05)	1.69 (1.42–2.03)	1.63 (1.33–2.07)	*0.014*
RBP < 0.70 μmol/L ^2^	0.7%	0.7%	0.7%	*NS*
RBP < 1.05 μmol/L ^2^	5.0%	3.1%	7.0%	*0.002*
Corrected ^1^ RBP (μmol/L; median IQR)	1.69 (1.39–2.10)	1.76 (1.45–2.10)	1.67 (1.36–2.11)	*0.012*
RBP < 0.70 μmol/L ^2^	0.6%	0.6%	0.5%	*NS*
RBP < 1.05 μmol/L ^2^	4.7%	2.9%	6.5%	*0.003*
Inflammation ^3^				
Normal	85.7%	84.3%	86.2%	*NS*
Incubation	3.9%	3.9%	3.6%	*NS*
Early convalescence	3.1%	2.8%	3.2%	*NS*
Late convalescence	7.6%	9.0%	6.9%	*NS*

^1^ Values corrected for inflammation as described in methods section. sTfR = soluble Transferrin Receptor; RBP = retinol Binding Protein, TBI = Total Body Iron; ^2^ Estimated prevalence, corrected for sampling method; ^3^ Normal = CRP ≤ 5 mg/L and AGP ≤ 1 g/L; Incubation = CRP > 5 mg/L and AGP ≤ 1 g/L. Early convalescence = CRP > 5 mg/L and AGP > 1 g/L; Late convalescence = CRP ≤ 5 mg/L and AGP > 1 g/L; ^4^ *p* value for difference between urban and rural populations, using binary logistic analysis for difference in prevalence and analysis of covariance (ANCOVA), controlling for age and sampling.

**Table 2:** Prevalence of low iron stores, iron-depleted erythropoeisis and vitamin A deficiency by region ^1^.

Indicator	Phnom Penh	South-East	South-West	West	North
Ferritin ^2^					
<15 μmg/L (%)	7.9%	6.8%	6.6%	8.0%	11.3%
<50 μmg/L (%)	32.4% ^a^	37.3% ^a,b^	36.6% ^a,b^	44.0% ^b^	44.8% ^b^
sTfR					
>8.3 mg/L (%)	8.1% ^a^	9.7% ^a^	5.2% ^a^	8.4% ^a^	18.0% ^b^
TBI					
<0 mg/kg (%)	4.0% ^a,b^	4.9% ^a,b^	3.5% ^a^	5.2% ^a,b^	8.4% ^b^
RBP ^2^					
<0.70 μmol/L	0.2%	0.3%	0.4%	0.7%	0.9%
<1.05 μmol/L	4.0%	6.6%	4.6%	7.6%	4.8%

^1^ Rows with different superscripts differ significantly (ANCOVA adjusted for age and sampling method, with Bonferroni correction for multiple comparisons, *p* < 0.05); ^2^ Values corrected for inflammation as described in methods section.

## References

[1] World Health Organization (2001). Iron Deficiency Anaemia Assessment, Prevention and Control. A Guide for Programme Managers.

[2] Kassebaum N.J., Jasrasaria R., Naghavi M., Wulf S.K., Johns N., Lozano R., Regan M., Weatherall D., Chou D.P., Eisele T.P. (2014). A systematic analysis of global anemia burden from 1990 to 2010. Blood.

[3] Perignon M., Fiorentino M., Kuong K., Burja K., Parker M., Sisokhom S., Chamnan C., Berger J., Wieringa F.T. (2014). Stunting, poor iron status and parasite infection are significant risk factors for lower cognitive performance in Cambodian school-aged children. PLoS ONE.

[4] Beard J.L. (2008). Why iron deficiency is important in infant development. J. Nutr..

[5] Burke R.M., Leon J.S., Suchdev P.S. (2014). Identification, prevention and treatment of iron deficiency during the first 1000 days. Nutrients.

[6] Stoltzfus R.J. (2011). Iron interventions for women and children in low-income countries. J. Nutr..

[7] Foote E.M., Sullivan K.M., Ruth L.J., Oremo J., Sadumah I., Williams T.N., Suchdev P.S. (2013). Determinants of anemia among preschool children in rural, western Kenya. Am. J. Trop. Med. Hyg..

[8] Suharno D., West C.E., Muhilal, Karyadi D., Hautvast J.G. (1993). Supplementation with vitamin A and iron for nutritional anaemia in pregnant women in West Java, Indonesia. Lancet.

[9] Koury M.J., Ponka P. (2004). New insights into erythropoiesis: The roles of folate, vitamin B12, and iron. Annu. Rev. Nutr..

[10] Weatherall D.J., Clegg J.B. (2001). Inherited haemoglobin disorders: An increasing global health problem. Bull. World Health Organ..

[11] Tsang B.L., Sullivan K.M., Ruth L.J., Williams T.N., Suchdev P.S. (2014). Nutritional status of young children with inherited blood disorders in western Kenya. Am. J. Trop. Med. Hyg..

[12] Cambodia Demographic and Health Survey 2014. https://dhsprogram.com/pubs/pdf/FR312/FR312.pdf.

[13] Karakochuk C.D., Whitfield K.C., Barr S.I., Lamers Y., Devlin A.M., Vercauteren S.M., Kroeun H., Talukder A., McLean J., Green T.J. (2015). Genetic hemoglobin disorders rather than iron deficiency are a major predictor of hemoglobin concentration in women of reproductive age in rural prey veng, Cambodia. J. Nutr..

[14] Mao B., Chheng K., Wannemuehler K., Vynnycky E., Buth S., Soeung S.C., Reef S., Weldon W., Quick L., Gregory C.J. (2015). Immunity to polio, measles and rubella in women of child-bearing age and estimated congenital rubella syndrome incidence, Cambodia, 2012. Epidemiol. Infect..

[15] Luman E.T., Worku A., Berhane Y., Martin R., Cairns L. (2007). Comparison of two survey methodologies to assess vaccination coverage. Int. J. Epidemiol..

[16] Erhardt J.G., Estes J.E., Pfeiffer C.M., Biesalski H.K., Craft N.E. (2004). Combined measurement of ferritin, soluble transferrin receptor, retinol binding protein, and C-reactive protein by an inexpensive, sensitive, and simple sandwich enzyme-linked immunosorbent assay technique. J. Nutr..

[17] Dallman P.R. (1986). Biochemical basis for the manifestations of iron deficiency. Annu. Rev. Nutr..

[18] Zimmermann M.B. (2008). Methods to assess iron and iodine status. Br. J. Nutr..

[19] Cook J.D., Flowers C.H., Skikne B.S. (2003). The quantitative assessment of body iron. Blood.

[20] Dijkhuizen M.A., Wieringa F.T., West C.E., Muherdiyantiningsih, Muhilal (2001). Concurrent micronutrient deficiencies in lactating mothers and their infants in Indonesia. Am. J. Clin. Nutr..

[21] De Pee S., Dary O. (2002). Biochemical indicators of vitamin A deficiency: Serum retinol and serum retinol binding protein. J. Nutr..

[22] Wieringa F.T., Dijkhuizen M.A., West C.E., Northrop-Clewes C.A., Muhilal (2002). Estimation of the effect of the acute phase response on indicators of micronutrient status in Indonesian infants. J. Nutr..

[23] Thurnham D.I., McCabe L.D., Haldar S., Wieringa F.T., Northrop-Clewes C.A., McCabe G.P. (2010). Adjusting plasma ferritin concentrations to remove the effects of subclinical inflammation in the assessment of iron deficiency: A meta-analysis. Am. J. Clin. Nutr..

[24] Thurnham D.I., McCabe G.P., Northrop-Clewes C.A., Nestel P. (2003). Effects of subclinical infection on plasma retinol concentrations and assessment of prevalence of vitamin A deficiency: Meta-analysis. Lancet.

[25] Thurnham D.I., Northrop-Clewes C.A., Knowles J. (2015). The use of adjustment factors to address the impact of inflammation on vitamin A and iron status in humans. J. Nutr..

[26] Milman N. (2011). Iron in pregnancy: How do we secure an appropriate iron status in the mother and child?. Ann. Nutr. Metab..

[27] Tanumihardjo S.A. (2004). Assessing vitamin A status: Past, present and future. J. Nutr..

[28] George J., Yiannakis M., Main B., Devenish R., Anderson C., An U.S., Williams S.M., Gibson R.S. (2012). Genetic hemoglobin disorders, infection, and deficiencies of iron and vitamin A determine anemia in young Cambodian children. J. Nutr..

[29] Theary C., Panagides D., Laillou A., Vonthanak S., Kanarath C., Chhorvann C., Sambath P., Sowath S., Moench-Pfanner R. (2013). Fish sauce, soy sauce, and vegetable oil fortification in Cambodia: Where do we stand to date?. Food Nutr. Bull..

[30] Van Thuy P., Berger J., Nakanishi Y., Khan N.C., Lynch S., Dixon P. (2005). The use of NaFeEDTA-fortified fish sauce is an effective tool for controlling iron deficiency in women of childbearing age in rural Vietnam. J. Nutr..

[31] Semba R.D., de Pee S., Panagides D., Poly O., Bloem M.W. (2003). Risk factors for nightblindness among women of childbearing age in Cambodia. Eur. J. Clin. Nutr..

